# Supplementary material for: EZH1 and EZH2 promote skeletal growth by repressing inhibitors of chondrocyte proliferation and hypertrophy
Source: Nat Commun. 2016 Nov 29;7:13685. doi: 10.1038/ncomms13685 (PMC5477487; doi:10.1038/ncomms13685)
Supplement: Supplementary Information — Supplementary Figures 1-11 and Supplementary Table 1. [file ncomms13685-s1.pdf]

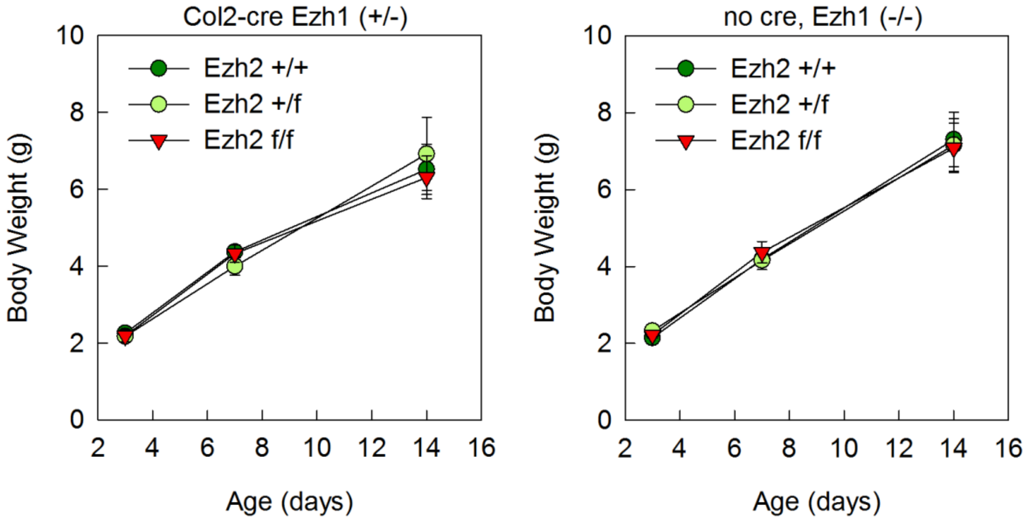

Supplementary Figure 1 Growth curves of Ezh2 wildtype, cartilage-specific heterozygotes, and cartilage-specific homozygous knockout mice, all with a Col2-cre, Ezh1+/- background (left panel). Growth curves of Ezh2 wildtype, cartilage-specific heterozygotes, and cartilage-specific homozygous knockout mice, all with an Ezh1-/- background (right panel).

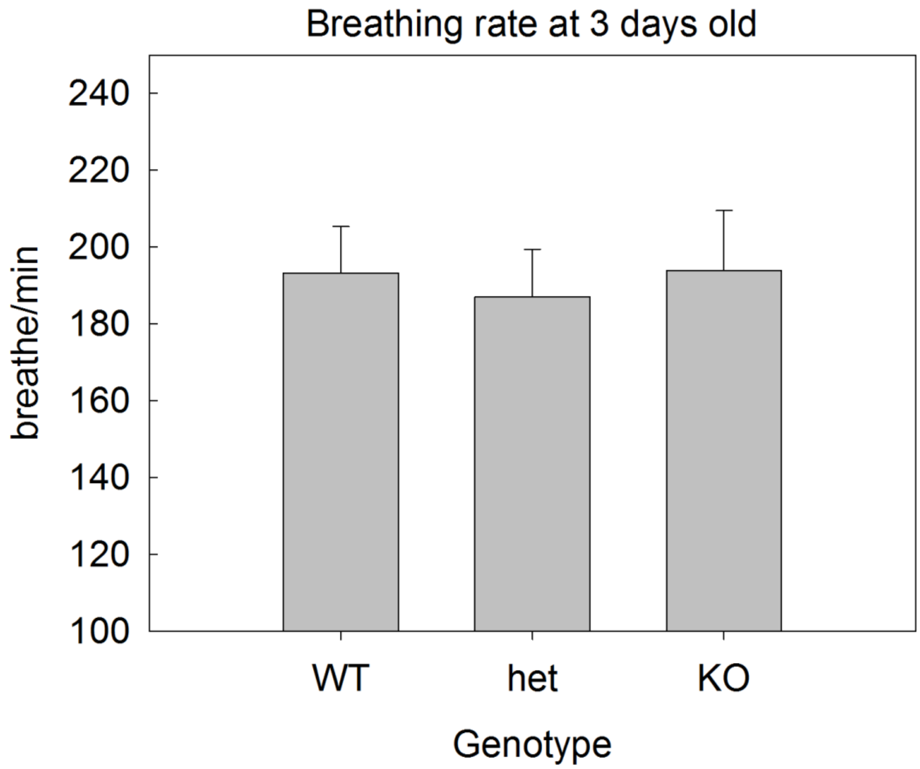

Supplementary Figure 2 Immunohistochemistry for Ezh1 (brown color) of tibial growth plate from mice with a Ezh1 -/- background (left) or Ezh1 +/- background (right). Lower panels showed the higher magnification images of PZ in the growth plate.

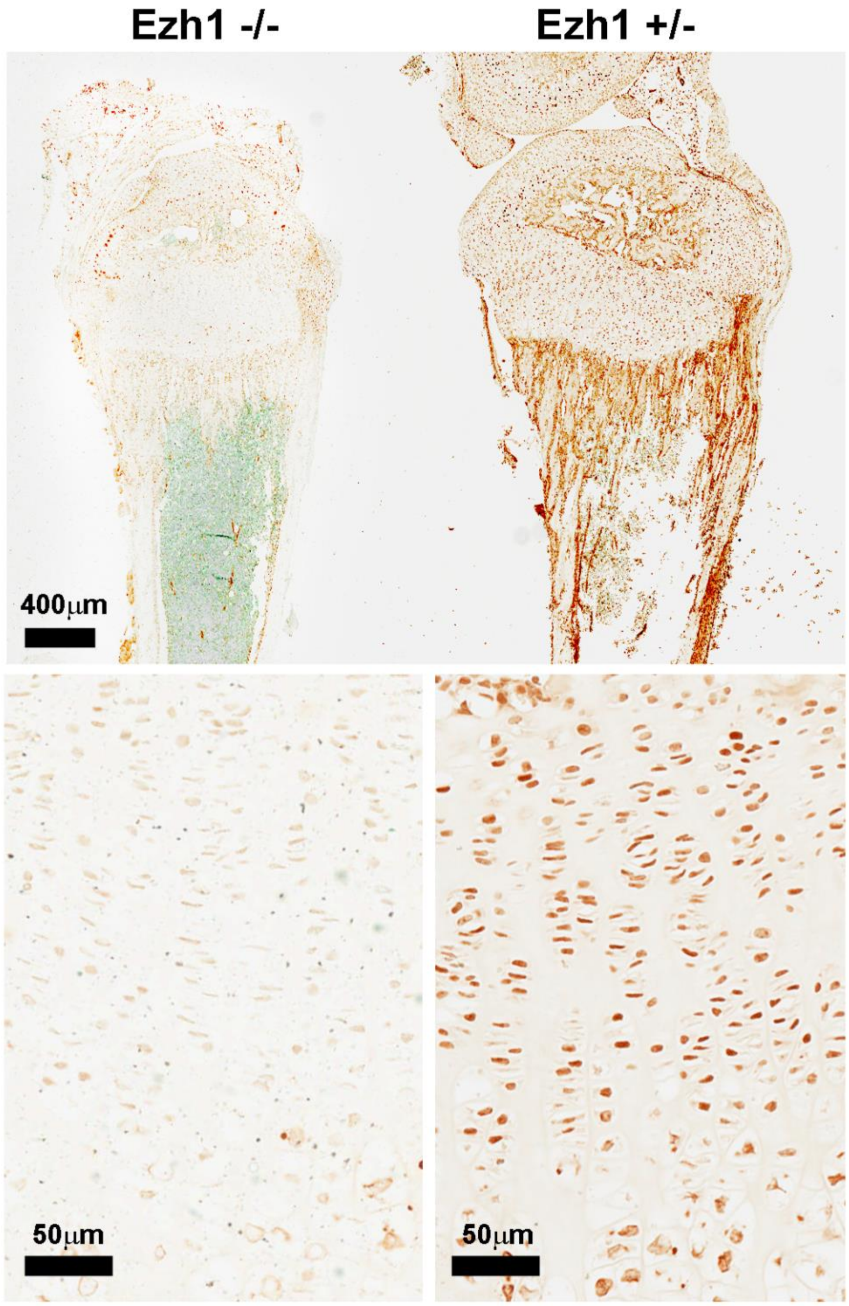

Supplementary Figure 3 Respiratory rate of 3-day old Ezh2 wildtype, cartilage-specific heterozygotes, and cartilage-specific knockout mice, all with an Ezh1-/- background.

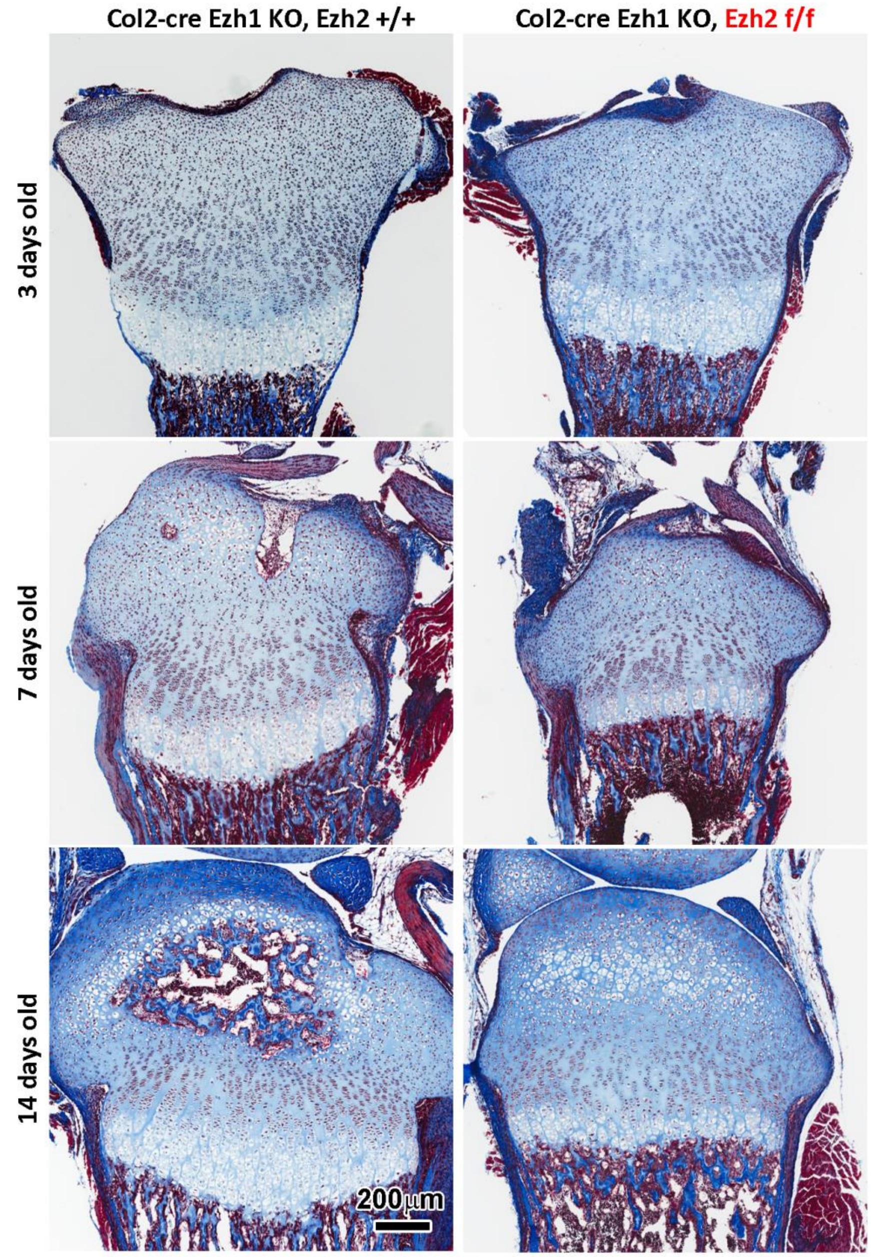

Supplementary Figure 4 Images of whole epiphysis of the proximal tibia taken from Ezh1/2 mice (right panels) and wild-type littermates (left panels) at different ages.

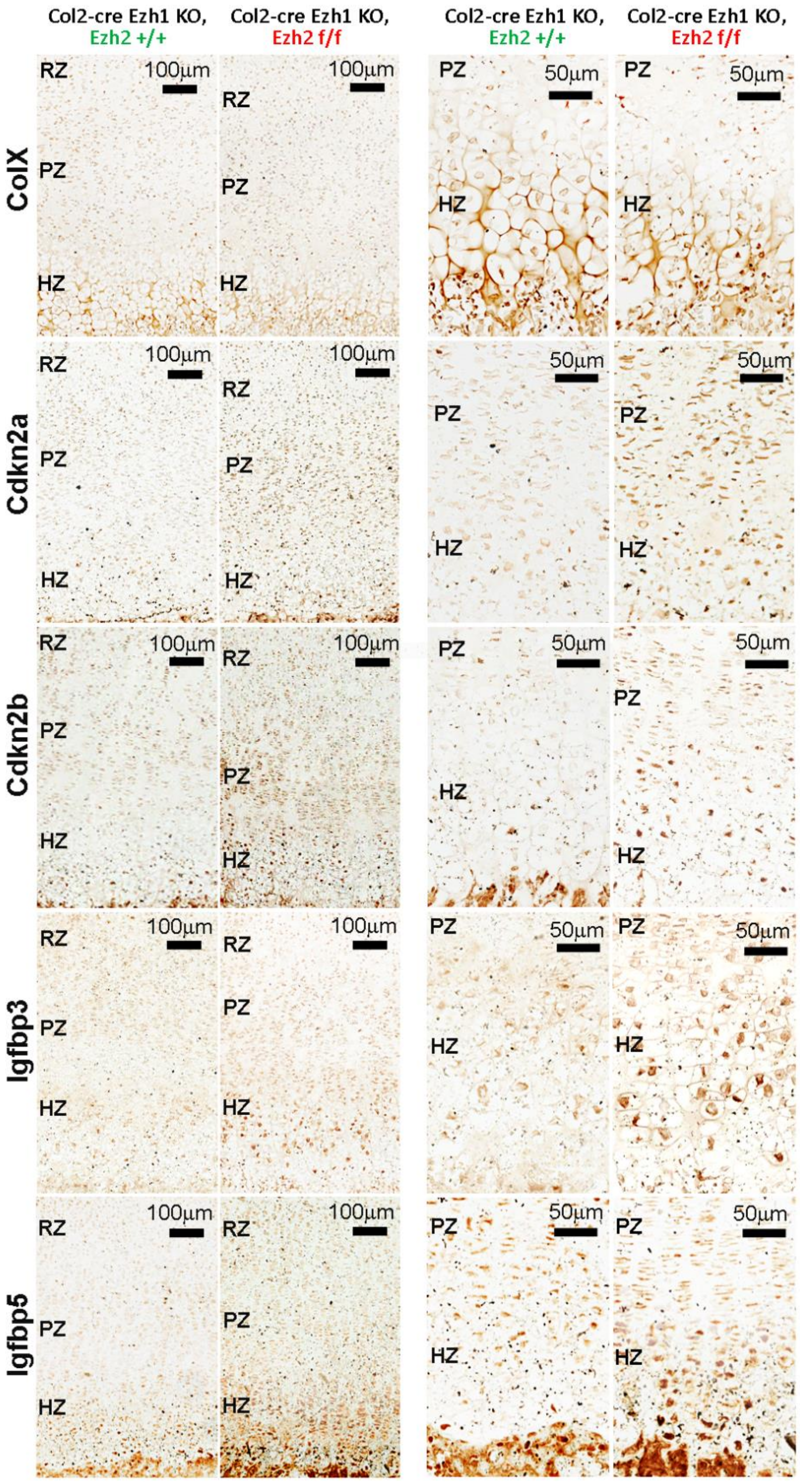

Supplementary Figure 5 Immunohistochemistry for ColX, Cdkn2a, Cdkn2b, Igfbp3, and Igfbp5 of 3 day old tibial growth plate from Ezh1/2 mice and wild-type littermates. Higher

magnification images were shown on the right. RZ, resting zone; PZ, proliferative zone; HZ, hypertrophic zone.

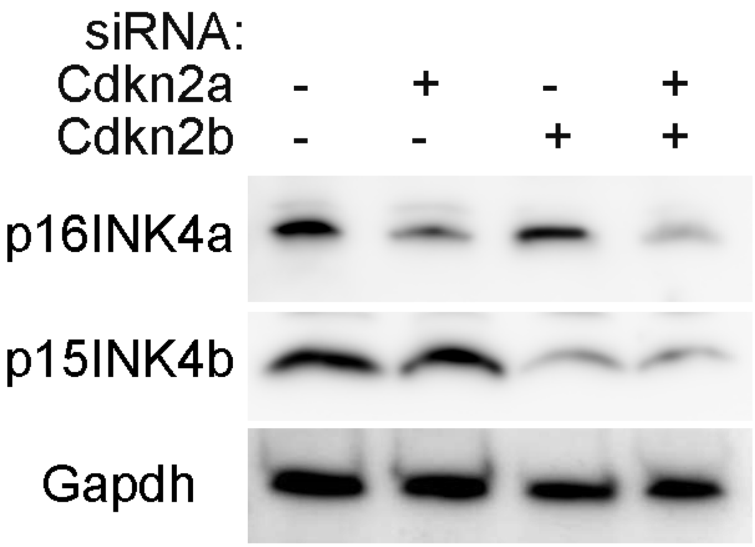

Supplementary Figure 6 Western blot showing decreased protein levels of $p16^{INK4a}$ (Cdkn2a) and $p15^{INK4b}$ (Cdkn2b) after transfection with siRNA. Gapdh was used as an internal control.

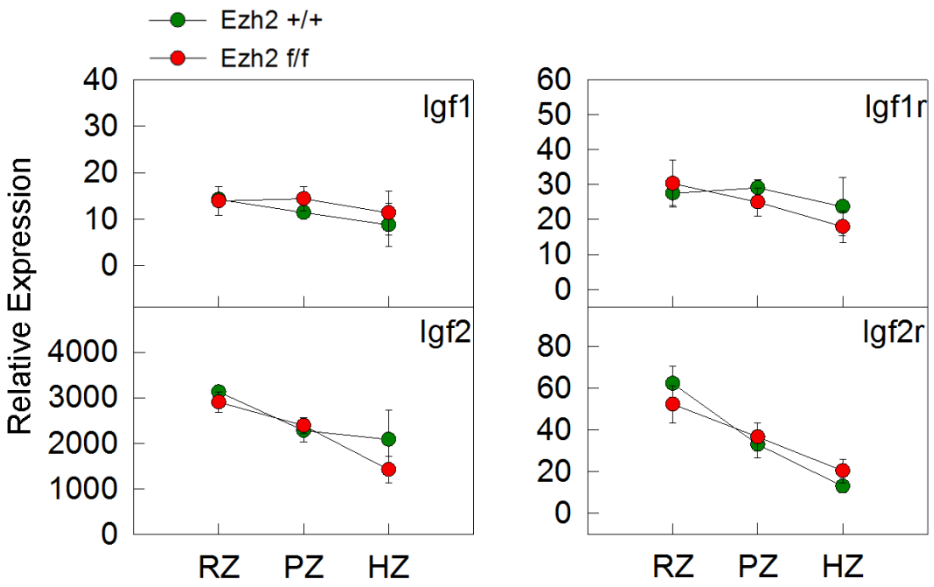

Supplementary Figure 7 Relative expression of Igf1, Igf2, Igf1r, and Igf2r in different zones (RZ, PZ, HZ) isolated from proximal tibial growth plates of 3-day old Ezh1-/- that are wildtype (Ezh2 +/+) or homozygous (Ezh2 fl/fl) for cartilage-specific Ezh2 knockout. Tissue was isolated by LCM and mRNA measured by quantitative real-time PCR.

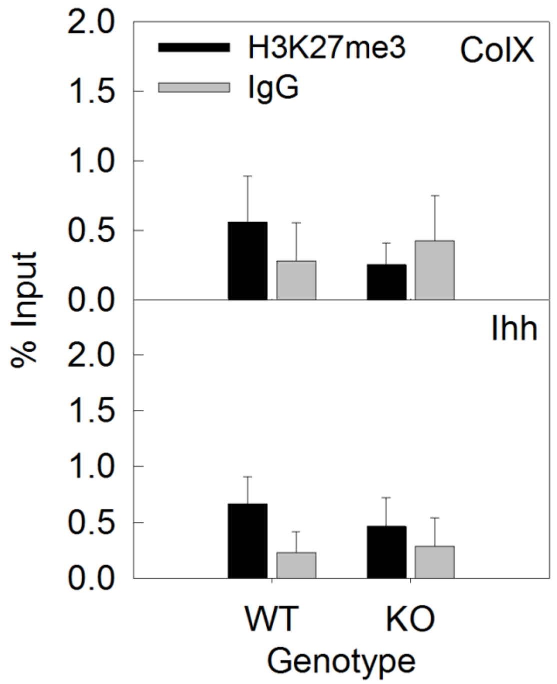

Supplementary Figure 8 Chromatin immunoprecipitation (ChIP) with H3K27me3 antibody (or IgG), followed by real-time PCR to compare levels of histone modification near transcription start site of Collagen X and Ihh, between chondrocytes isolated from 1-week old Ezh1-/- mice that are wildtype (WT) or homozygous (KO) for cartilage-specific Ezh2 knockout.

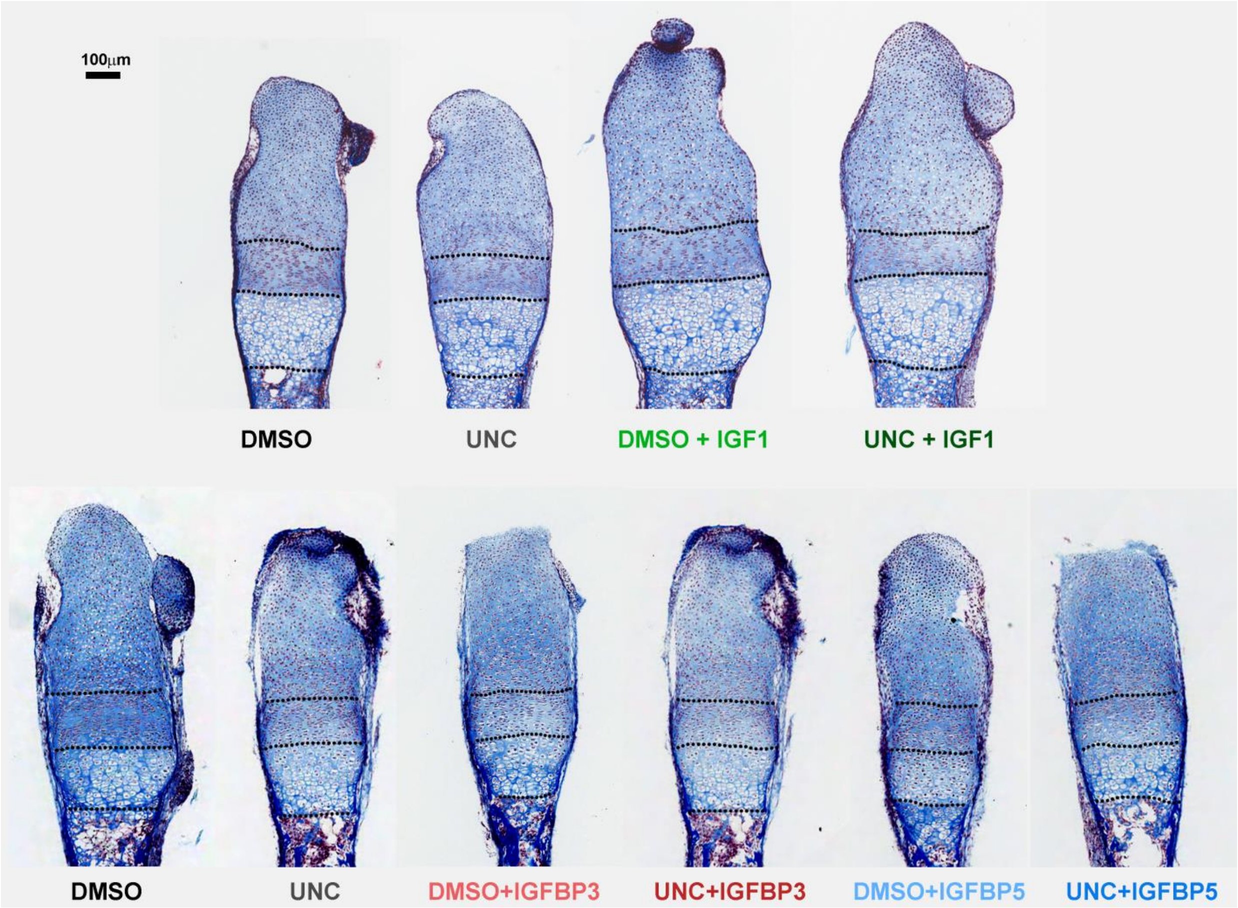

Supplementary Figure 9 Images of whole epiphysis of cultured fetal mouse metatarsal bone upon treatment of DMSO, Igf1, Igfbp3, Igfbp5, and/or Ezh1/2 inhibitor UNC1999. Close up images of the hypertrophic zones of these metatarsal bones were shown in Figure 4l and 4o.

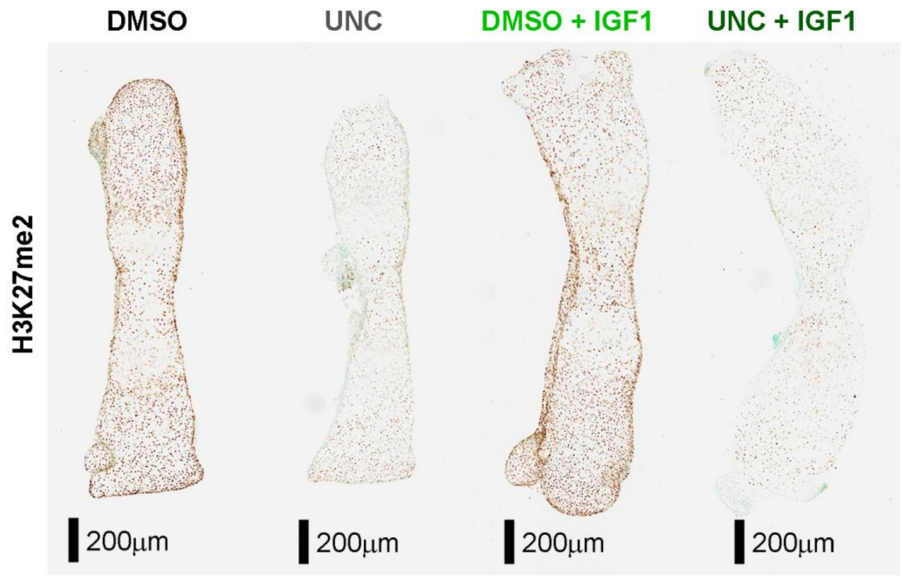

Supplementary Figure 10 Immunohistochemistry for H3K27me2 (brown color) of wildtype fetal mouse metatarsal bones treated with DMSO or UNC1999, with or without Igf1.

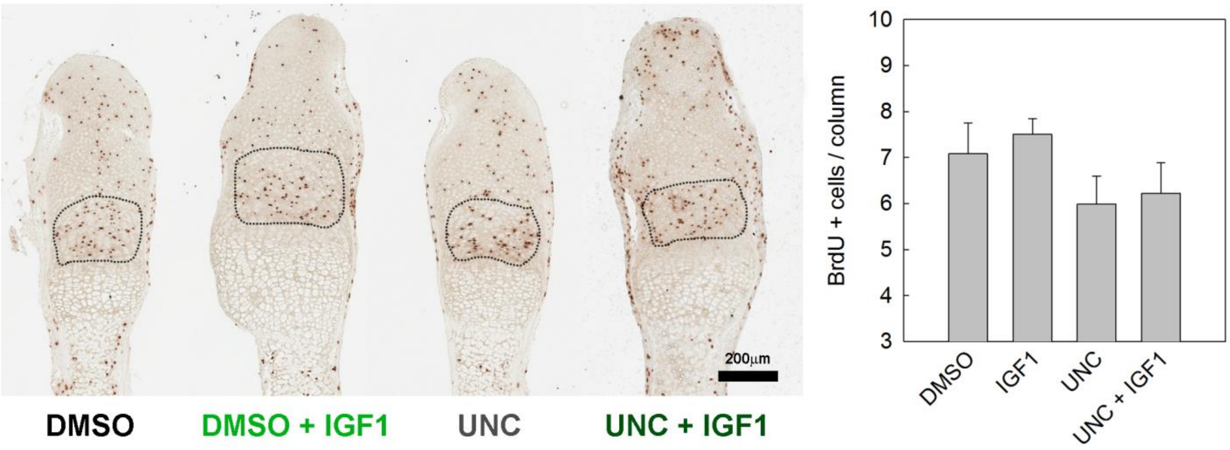

Supplementary Figure 11 BrdU stained fetal metatarsal bone treated with DMSO or UNC1999, with or without Igf1. Number of BrdU positive chondrocytes in the PZ (indicated by the dotted line) were counted and averaged between 5 bones. Igf1 treatment only showed a slight tendency of increased proliferation (not statistically significant).

Supplementary Table 1. Ezh2 genotype frequency (under Col2cre, Ezh1-/- background)

	Number of newborns	Observed percentage	Expected percentage
Wildtype (+/+)	155	30.0%	25%
Heterozygous (+/f)	291	56.3%	50%
Knockout (f/f)	71	13.7%	25%
